# Feasibility and Acceptability of the Living My Life Program for Rural and Remote Stroke Survivors

**DOI:** 10.1111/ajr.70166

**Published:** 2026-04-07

**Authors:** Sarah M. Jackson, Lucy M. Byrnes, Shaun D. Solomon, Bridee A. Neibling, Louise F. Brown, Ian M. Atkinson, Ruth N. Barker

**Affiliations:** ^1^ College of Healthcare Sciences James Cook University Townsville Queensland Australia; ^2^ Murtupuni Centre for Rural and Remote Health James Cook University Mount Isa Queensland Australia; ^3^ Stroke Survivor Australia; ^4^ Chancellery James Cook University Townsville Queensland Australia; ^5^ The Cairns Institute James Cook University Cairns Queensland Australia

**Keywords:** acquired brain injury, online, recovery, regional, rehabilitation, technology, telerehabilitation

## Abstract

**Objective:**

Determine feasibility and acceptability of the Living My Life Program from the perspective of rural and remote stroke survivors and identify refinements required.

**Setting:**

Queensland, Australia.

**Participants:**

Adults diagnosed with stroke > 6 months prior, living at home, and willing to use videoconferencing technology.

**Design:**

Pragmatic mixed methods multiple case study design, incorporating elements of grounded theory and action research.

**Intervention:**

Twelve‐week online program designed to support stroke survivors to recover their way, in their world, using technology.

**Main Outcome Measure(s):**

Feasibility (Q1‐3) and acceptability (Q4) were assessed according to minimum success criteria to answer the following key questions: were stroke survivors able to: (Q1) participate in the Program as intended? (Q2) use technology to participate? (Q3) measure their progress according to what mattered to them? and (Q4) did stroke survivors find the Program acceptable?

**Results:**

Four of 5 participants completed the Program using everyday technologies; one did not complete due to illness. Participants measured their progress using the Patient‐Specific Functional Scale, with improvement reported in ≥ 1 goal(s). The Program was highly acceptable (Acceptability of Intervention Measure: median 20/20, range 18–20). Participants felt they could fit the Program into their lives easily, enabling them to drive their recovery and make progress towards what mattered to them. Refinements were incorporated during piloting or noted for future development.

**Conclusion:**

The Living My Life Program was feasible and acceptable from the rural and remote stroke survivors' perspective. The next step is to offer the Program to stroke survivors from diverse cultures and with varying stroke‐related impairments and severity to determine its broader applicability.

**Trial Registration:**

ClinicalTrials.gov identifier number: ACTRN12622000967785

## Abstract (highlights)

**What is already known on this subject?:**

- Rural and remote stroke survivors in Australia have limited opportunities to participate in rehabilitation. When they do participate, it generally disrupts their lives.
- They are open to using technology when it works for them in their lives.
- Use of technology to participate in rehabilitation is often suggested yet rarely evaluated from the perspective of rural and remote stroke survivors.

**What does this study add?:**

- Rural and remote stroke survivors were able to participate in rehabilitation using everyday technologies.
- Stroke survivors felt the 12‐week online Living My Life Program was acceptable because they made progress towards what mattered to them, without disrupting their lives.
- Further research is needed to determine the Program's broader applicability with stroke survivors of diverse cultures, with varying impairments and severity, and earlier in the recovery journey.

## 1 Introduction

Rural and remote stroke survivors' opportunities to participate in rehabilitation are restricted. In Australia, poor access to local services, long distances to stroke rehabilitation services, and little or no continuity of care make participating in rehabilitation difficult [1, 2, 3]. Rural and remote stroke survivors are looking for ways to continue their recovery where they live [4], which may include using technology to support them [5, 6].

Using technology to overcome challenges posed by remoteness has been recommended [7, 8], and is supported financially in Australia, e.g., through the National Disability Insurance Scheme (NDIS) [9]. Although rural and remote stroke survivors are open to using technology [2], its use has been minimally investigated from their perspective [10]. Further research is needed to understand how stroke survivors can harness technology to continue to recover in their local community.

The Living My Life Program (‘Program’) [11] was designed to support rural and remote stroke survivors to continue to recover where they live, using technology. This Program was developed in response to a series of interviews with stroke survivors that explored their lens on recovering: they wanted to recover their way, in their world, while living their life, as it evolves [2]. This lens was combined with evidence for supporting stroke survivors to continue recovering where they live [10, 12], using technology [13] and supported by a coach remotely [14, 15, 16].

The aim of this study was to pilot the Living My Life Program with rural and remote stroke survivors to explore and learn from their real‐life experiences. The objectives were to assess the Program's feasibility and acceptability from the perspective of stroke survivors and to make recommendations for refinement prior to broader implementation. There were four key questions to be answered about the Program: were stroke survivors able to (1) participate as intended? (2) use technology to participate? (3) measure their progress according to what mattered to them? and (4) did stroke survivors find the Program acceptable?

## 2 Methods

### 2.1 Design

A pragmatic mixed methods multiple case study design incorporating elements of grounded theory and action research formed the overarching methodology. A pragmatic approach [17] allows methods to be adapted to the research context [18], to ensure actionable outcomes are ready for real‐world use [19]. Hence, methods were adapted to enable diversity in stroke survivors' circumstances to be accommodated and expertise and resources in the rural and remote setting to be leveraged. Case study design, applied with a small group of stroke survivors, provided flexibility to tailor each individual's program to their real‐life context [20], accommodating diversity in their demographics, locations, stroke‐related impairments and recovery goals. Through a series of sequential overlapping case studies, each stroke survivor formed a single case bounded by the duration of their program (12 weeks). Narratives detailing each stroke survivor's experience are reported separately [11].

An action research approach, involving staggering of each stroke survivor's commencement date, allowed refinements to be identified, integrated and piloted with subsequent participants, or noted for ongoing development [21]. Elements of grounded theory which underpinned Program development [2], were similarly incorporated to ensure the Program stayed true to its roots. The lead researcher continually refined the Program in response to stroke survivors' feedback and in consultation with a stroke survivor co‐researcher and an expert panel [21] with cultural, technological and rehabilitation expertise in rural and remote settings. For example, a participating stroke survivor suggested introducing a goal‐setting worksheet before the first session, which was implemented with a subsequent stroke survivor. Program development, including refinements, will be reported separately.

By choosing mixed methods, the stroke survivors' perspective could be measured using quantitative methods, interpreted using qualitative methods [22] and integrated to determine feasibility and acceptability of the Program from the stroke survivors' perspective.

The trial was approved by the James Cook University Human Research Ethics Committee (H8616) and registered with the Australian New Zealand Clinical Trials Registry (ACTRN12622000967785).

### 2.2 Positionality

The lead researcher (SJ) is a female clinical and research physiotherapist experienced in providing community rehabilitation and telehealth services in remote locations. SJ knew three of the stroke survivors who volunteered from community rehabilitation; two of whom were previous interviewees [2]. Based on her understanding of the context, SJ chose a pragmatic approach to ensure the Program would be useful for stroke survivors in their real‐world settings. Accordingly, when staff turnover prevented implementation within a local service, SJ became the allied health coach who delivered the Program online while concurrently continuing as the researcher who collected and analysed the data. Similarly, as coach, SJ opportunistically met once with three stroke survivors individually in person when they were simultaneously in the same community. This dual role enabled SJ, who identifies with the constructivist paradigm [23], to develop a deeper understanding of the stroke survivors' experiences, from their own perspective.

The stroke survivor co‐researcher (LMB) is a female psychologist with lived experience of stroke. LMB contributed to the Program foundation as an interview participant, to Program development as an expert panellist, and to the Program pilot as the first participant. To minimise the influence of the views of other expert panellists on LMB's perspective, LMB's case study data were collected before the first expert panel meeting. Recurring insights from LMB helped ensure the Program content and delivery stayed true to the stroke survivors' lens.

The expert panel was female allied health clinicians and researchers (BN, LFB, RB), a male cultural mentor (SS), and a male technology researcher (IA); all of whom had experience in rural and remote settings. Panellists contributed to Program development through individual and group meetings with the lead researcher on a routine and ad‐hoc basis.

### 2.3 Participants

Five stroke survivors were recruited in keeping with case study design which emphasises in‐depth exploration of a small number of cases [22]. Purposive sampling was used to identify participants of varying age, gender, location, cultural identity, time since stroke (Table 1) and aspirations for recovery. Inclusion criteria were adults (> 18 years old) with a diagnosis of stroke > 6 months ago; living at home; who were able to communicate by expressing themselves in their preferred way (which may include augmented methods or devices), or have a significant other facilitate communication; communicate via videoconference; and provide informed consent. Stroke survivors participating in centre‐based rehabilitation at the same time were excluded.

**TABLE 1:** Participant characteristics (*n* = 5).

Characteristic		Mean, median (range)	*n* (%)
**Age**, years		54.2, 52 (37–78)	
**Time since stroke**, years		7.4, 7 (0.67–14)	
**Stroke Impact Scale** ^a^, recovery (/100)		66, 73 (40–80)	
**Gender**			
Female			2 (40)
Male			3 (60)
**Cultural identity**			
Aboriginal			1 (20)
Non‐indigenous			4 (80)
**Rurality** ^b^			
MM5 – small rural town			2 (40)
MM6 – remote community			2 (40)
MM7 – very remote community			1 (20)
**Industry**			
Health			1 (20)
Mining			1 (20)
Transport			2 (40)
Volunteer			1 (20)

^a^ *n* = 4.

^b^ Modified monash model [24].

Stroke survivors were recruited from rural and remote Queensland (MM3–7) [24]. Recruitment occurred via a flyer distributed to community organisations, health professional networks, support groups, previous research participants (with consent), social media and radio, from July 2022 to May 2023. All interested stroke survivors were screened via phone by an individual who was independent of the research. Eligible stroke survivors were provided with an explanation of the study and an information and consent form; if they chose to participate, they were required to return the completed form via their preferred method (email, in‐person, post). To recruit stroke survivors who identified as Aboriginal and Torres Strait Islander, the lead researcher worked through this recruitment process with cultural mentors and an Aboriginal community‐controlled health service and offered interested stroke survivors the option of support from a cultural mentor.

### 2.4 The Living My Life Program Prototype

The Program prototype [11] was a 12‐week individual program conducted via Zoom with the coach for at least 9 × 1‐h sessions. Weekly online meetings were scheduled according to individual preferences (day, time, venue). Intervals between sessions increased as the participant progressed through the Program, to encourage their confidence with managing their plan. The participant was guided by the coach through 5 stages (Table 2): (1) work out what matters; (2) co‐design the action plan; (3) trial the action plan; (4) fit the plan into life; and (5) review the plan and progress [11]. The coach utilised goal setting [14], motivational interviewing [15] and action planning [16] techniques to actively engage the participant in planning and monitoring their progress. Each participant chose the technologies they used, and the support they needed, according to their way (e.g., routines or preferences) and in their world (e.g., resources available). To gain clinical and technological knowledge around participants' goals and chosen technologies, the coach scanned current evidence and sought guidance from clinical, technological and cultural experts (e.g., the coach met with an author of a recent study targeting handwriting [25]). The coach also reviewed participants' sessions and their technology use, then discussed the findings with the expert panel [26].

**TABLE 2:** Stages of the Living My Life Program prototype [11].

Stage	What's involved
1. Work out what matters	‐ Interview to understand what matters to participant (goals and baseline measures), their way and their world (e.g., strengths to draw on; limitations to accommodate), including technology.
2. Co‐design the action plan	‐ Determine activities: goal‐practice (e.g., walking around town); part‐practice (e.g., treadmill); and address impairments (e.g., knee strengthening). ‐ Explore technologies and support needed. ‐ Assess confidence with plan and discuss strategies.
3. Trial the action plan	‐ Trial activities, modify with coaching. ‐ Technology use supported as needed.
4. Fit the plan into life with coaching	‐ Complete activities; modify to fit their life, their way, or their world. ‐ Record log.
5. Review plan and progress	‐ Review goals, progress, and plan; adjust accordingly. ‐ Summarise in manual.

### 2.5 Assessment of Feasibility and Acceptability

Feasibility (Q1‐3) and acceptability (Q4) were firstly assessed against minimum success criteria (i.e., lowest threshold indicative of success [27]), then explained qualitatively for each key question.

Data were either obtained via self‐report by participants, or comprised real‐time observations and reflections recorded by the lead researcher. Participant data, listed in Table 3, were recorded via the recruitment log, screening sheets, participant logs, session and interview video‐recordings, and surveys. These were collected using phone calls, text messages, emails, online surveys, and videoconferences. Researcher observations and reflections were recorded in a research journal.

**TABLE 3:** Participant data collected.

Demographic	Stroke‐related	Technology‐related	Program^b^	Outcomes	Acceptability^c^
Age^a^ Gender^a^ Ethnicity^a^ Location^a^ Occupation^b^ Funding^a^	Time since stroke^a^ Communication preferences^a^ Aspirations for recovery^a^ Self‐assessed function and recovery (SIS)^b^	Availability^a^ Use^b^ Preferences^b^ Support^b^	Attendance Completion Adverse events Compliance Adherence Satisfaction with activities completed Session feedback	Goal performance (PSFS)^b^, ^d^ Satisfaction with performance^b^, ^d^ Perceived change (PGIC)^c^	Survey (AIM) Semi‐structured interview

Abbreviations: AIM, Acceptability of Intervention Measure [28]; PGIC, Patient Global Impression of Change [29]; PSFS, Patient‐Specific Functional Scale [30]; SIS, Stroke Impact Scale v3.0 [31].

^a^ Collected at recruitment, by clinician or research assistant.

^b^ During the program.

^c^ After the program, by lead researcher.

^d^ Weeks 1, 4, 8 and 12; and when a new goal was set.

Data collection instruments that would prioritise the participant's perspective and were easy to use were utilised [17, 32]. Patient‐Reported Outcome Measures (PROMs) with demonstrated validity and reliability were selected, or measures were adapted from a scale already in use to maintain consistency, increasing the ease of completion and therefore reliability. Measures included: goal performance, using the Patient‐Specific Functional Scale (PSFS) [30]; satisfaction with performance, and with activities completed, using the scale 0 = ‘not at all satisfied’ and 10 = ‘completely satisfied’; and perceived change, using the Patient Global Impression of Change (PGIC) [29]. Satisfaction with activities completed is a measure that was added during piloting with the first participant after finding that their attitude towards activities completed was influenced by what was happening in their life at the time. Program acceptability was assessed using the Acceptability of Intervention Measure (AIM) [28], followed by a semi‐structured, individual interview (mean duration: 88 min; range: 64–131 min) conducted on Zoom by the lead researcher with participants at the end of their program (File S1). Qualitative data from the lead researcher's journal were used to contextualise participant data. Quantitative data were entered into Excel and qualitative data were imported into NVivo (QSR International Pty Ltd) in preparation for analysis.

### 2.6 Data Analysis

For each research question, quantitative and qualitative data were analysed separately, then integrated and reported in a table. Quantitative data were analysed descriptively and presented using raw counts (%) or the mean and median (range). Qualitative data relating to feasibility were coded deductively to identify barriers and facilitators. Qualitative data relating to acceptability were coded inductively [23] by the lead researcher. Similar codes were drawn into categories and discussed with a co‐researcher (RB) and the expert panel. Categories were then tested, including searching for negative cases, in subsequent readings of the data. Findings were reported by category with quotations showing how the category is grounded in participants' responses. Integration occurred by aligning the quantitative and qualitative findings in a table [33], and then comparing and contrasting the findings to answer the key questions.

Credibility was established through repeated engagement with the data and refining qualitative data collection to understand surprising or contradictory results, debriefing with co‐researchers, presenting qualitative and quantitative findings alongside integration, and including quotations. Audio‐visual summaries of emerging results were confirmed by participants during member‐checking prior to finalising analysis. The lead researcher reflected on research practices through discussion with the expert panel. Discussions, field notes, and memos were documented, and data records maintained to form an audit trail.

## 3 Results

The Program was feasible and acceptable when assessed against the minimum success criteria listed in Table 4. Integration of quantitative and qualitative findings is outlined in Table 4 and summarised for each key question below.

**TABLE 4:** Integration of quantitative and qualitative findings to answer the key questions.

Criteria	Minimum success	Quantitative summary *n* (%)	Qualitative summary	Integration
**Q1: Were stroke survivors able to participate in the Program as intended?**				
Reach	5 participants	12 people identified.	Recruited via local networks and social media. Support needed to engage Aboriginal and Torres Strait Islander Peoples, people with communication difficulties, and people in remote communities. Motivated to improve recovery for themselves and other stroke survivors.	Stroke survivors were able to participate when the Program fitted within their lives. Designing and moulding their plans helped. Seeing progress motivated them to continue.
		7 people screened, all eligible.		
		5 consented.		
Attendance^a^	80% completion	4/5 (80) completed.	Fitted amidst commitments (e.g., work, holidays) and life experiences (e.g., injury) because online and flexible. Getting started was difficult when limited connection with coach (e.g., internet, illness).	
		1/5 (20) did not complete.		
	80% attendance	35/36 (97) sessions attended^b^.		
		66min (30–149) duration^[Link]^, ^a^.		
Compliance^b^	80% logs	33/34 (97) logs.	Largely printed, handwritten, and submitted via text, email, or read aloud. Log also provided ‘to‐do list’ and plan for week ahead. Digital log may be easier for some and more accurate.	
Adherence and satisfaction^b^	80% completion	19/33 (58) completed.	Contributing to plan, seeing improvements, feeling accountable, finding time, and establishing habits and routines facilitated completion. Shifting priorities or motivations, emotions, circumstances (e.g., injury, travel), inability to perform activity (e.g., limited movement), and technology frustrations limited completion. Generally satisfied with achievements given circumstances.	
	80% satisfaction	8/10 (2.5–10) satisfied^d^.		
Adverse events^a^	Nil serious adverse events	Nil serious reported.	Continued amidst injuries and illnesses in daily lives. Modified plans by reducing load and/or prioritising activities. Sessions rescheduled (e.g., around appointments).	
		Adverse events unrelated to Program:		
		5/5 (100) illness: delayed start (1); did not complete (1); UTA session (1); attended as planned (2).		
		2/5 (40) injury: work‐related (1); fall + recurrence of pre‐existing injury (1).		
**Q2: Were stroke survivors able to use technology to participate in the Program?**				
Technology use^a^	80% used preferred technology	4/5 (80) used preferred technology to:	Chosen by purpose (e.g., practice writing), preferences (e.g., convenience, screen size), and availability (e.g., home, community). Technology generally worked well; set aside if wasn’t useful. Zoom comfortable for most, to build relationship and get feedback; ≥ 1hr challenging for some. In‐person complemented online sessions. For participant unable to participate at home due to poor internet connectivity, connection arranged at community service but not trialled prior to illness.	Stroke survivors used everyday technologies in their life to participate. Supported by people around them and the coach, when needed.
		‐ interact with coach (e.g., Zoom, email, text, phone);		
		‐ complete activities (e.g., online knitting videos, handwriting app);		
		‐ measure progress (e.g., performance videos, feedback).		
Availability^a^	80% use technologies available in their world	Available technologies:	Added technology as needed (e.g., camera, stylus). Occasional glitches resolved quickly.	
		5/5 (100) smartphone;		
		4/5 (80) laptop/computer;		
		4/5 (80) tablet/iPad;		
		1/5 (20) activity monitor.		
Support^a^	80% identify and meet support needs	2/5 (40) used Zoom independently;	Online and in‐person support (e.g., to login to Zoom) provided by family, friends, colleagues, health professional, care provider, or coach. Consider hybrid models when in‐person support required.	
		2/5 (40) used Zoom with support;		
		1/5 (20) unable to use Zoom.		
**Q3: Were stroke survivors able to measure their progress according to what mattered to them?**				
Goal setting^a^	80% set goal(s)	4/5 (80) set 1–3 goals.	Goals relevant in their daily lives. What matters emerged through discussion; shifted with progress, daily life, and time.	Stroke survivors measured their progress using PROMs complemented by feedback (e.g., before and after video) that they reported to find more meaningful.
	80% score goal(s) monthly on PSFS	4/5 (80) scored goals in Weeks 1,4,8 and 12.		
Progress^a^	80% report positive change	4/5 (80) positive change ≥ 1 goal(s). Average change^[Link]^, ^a^:	Seeing progress was useful (e.g., scores, videos). Responses influenced by own feelings, expectations, life circumstances, progress, and priorities. Progress in goals and in life led to exploring recovery potential and adjusting goalposts.	
		‐ Performance (PSFS): +3.3 (−0.5–8);		
		‐Satisfaction: +3.6 (−0.5–9).		
		Impression (PGIC)^b^:		
		6/8 (75) goals improved minimally, much or very much; 2/8 (25) no change;		
		4/4 (100) ‘living my life’ improved much or very much.		
**Q4: Did stroke survivors find the Program acceptable?**				
Acceptability^b^	80% acceptable	AIM^d^: 20/20 (18–20)	Fitted the Program in easily because it worked in their lifestyle and their space. Felt they were driving their recovery by owning their plan and putting in the effort. Resulted in progress towards what mattered to them and in living their life.	Highly acceptable because it worked for participants: they continued recovering without disrupting their lives, using technology.

Abbreviations: AIM, Acceptability of Intervention Measure^28^; min, minutes; PGIC, Patient Global Impression of Change^29^; PROMs, Patient‐Reported Outcome Measures; PSFS, Patient‐Specific Functional Scale^30^; UTA, unable to attend.

^a^ *n* = 5.

^b^ *n* = 4.

^c^ Mean (range).

^d^ Median (range).

## 4 Q1: Were Stroke Survivors Able to Participate in the Program as Intended?

Five participants enrolled in the Program, all of whom varied in age, gender, cultural identity, time since stroke, rurality, and industries in which they worked. One participant, an Aboriginal man from a remote community, was unable to complete the Program due to an unrelated illness. After consenting, the participant met with the coach on the phone (6 times), on his own (4 times) or with a support worker present (2 times); and, later, with the cultural mentor (once). These calls served to build a relationship; to learn about what mattered to him, his way and his world; and, to plan and trial coaching sessions using technology. The participant's first formal session was arranged and then rescheduled twice to accommodate illness, after which it did not proceed. Four participants completed the Program, with high attendance (i.e., online coaching sessions) and compliance (i.e., completion of logs). While adherence (i.e., completion of activities) was lower than planned at times, participants were generally satisfied with what they had achieved amidst the busy‐ness of their lives. Participants prioritised activities and modified plans to accommodate illness or injury unrelated to the Program. Nil serious adverse events related to the Program were reported. Hence, the stroke survivors were able to participate in the Program as intended, because they were able to fit it into their lives.

## 5 Q2: Were Stroke Survivors Able to Use Technology to Participate in the Program?

Participants who completed the Program used everyday technologies to interact with the coach, complete activities, and measure their progress. All participants had experience with technologies, most commonly smartphones, tablets or iPads, and laptops or computers. Four participants had not used Zoom before. Technologies chosen varied according to purpose, participant preference, and availability. Participants sourced additional technology as required. They drew on in‐person support from people around them, including family and health professionals; sometimes complemented by online support from the coach. Technologies that did not work for participants were set aside temporarily (e.g., waiting for assistance to download app) or indefinitely (e.g., difficulty operating with limited hand function). Hence, the stroke survivors were able to participate using everyday technologies and their everyday technology supports.

For the participant who did not complete the Program, limited internet connectivity at his home meant that he could not join Zoom sessions from his smartphone. Alternative videoconferencing at a community service was arranged at a time convenient for the participant, the support worker, the coach, the cultural mentor and the facility; however, the participant was unable to attend due to illness at that time. In essence, this participant was not able to use his everyday technologies and supports.

## 6 Q3: Were Stroke Survivors Able to Measure Their Progress According to What Mattered to Them?

Participants' goals varied, e.g., mobility, communication, and activities (Table 5). Participants were able to measure their goals and generally reported positive improvements of ≥ 2‐points (i.e., minimal important change [30]) on the PSFS in ≥ 1 goal(s) and in their lives. Use of a combination of PROMs to measure change within each goal, highlighted that the strength of improvement could vary across PROMs (e.g., a smaller change in performance could bring about a larger change in satisfaction). Participants felt that scoring goals periodically was useful, but seeing progress in other ways (e.g., before and after videos) was often described as more meaningful. Hence, the stroke survivors were able to measure their progress according to what mattered to them using PROMs, as well as using ways that they found to be more meaningful, often using everyday technologies.

**TABLE 5:** Participant goals (*n* = 4) and measures of performance, satisfaction, and impression of change.

ID	What matters (Goal)	Performance (PSFS),/10			Satisfaction with performance,/10			Impression of change (PGIC)
		*Start*	*End*	*Change* ^a^	*Start*	*End*	*Change* ^a^	
1	Knitting using both hands	2	10	↑8	6	8	↑2	Much improved
	Exercising daily to manage fatigue	6	8	↑2	4.5	10	↑5.5	Much improved
2	Writing legibly in a timely manner	6	7.5	↑1.5	4	7	↑3	Very much improved
	Climbing stairs without using handrails	4	6	↑2	3	6	↑3	No change
3	Walking 20 min without pain	3	5	↑2	2	7	↑5	Much improved
4	Using technology with confidence to communicate	2	8	↑6	0	9	↑9	Very much improved
	Singing higher notes in choir	3	8	↑5	5	7	↑2	Minimally improved
	Getting around home and community safely^b^	8	7.5	↓‐0.5	8	7.5	↓‐0.5	No change

Abbreviations: PGIC, Patient Global Impression of Change [29]; PSFS, Patient‐Specific Functional Scale [30].

^a^ End–Start.

^b^ Set in Week 4.

## 7 Q4: Did Stroke Survivors Find the Program Acceptable?

The four participants who completed the Program reported a median AIM score of 20/20 (File S2) indicating the Program was highly acceptable. These participants confirmed this during their final interview when they reported that they could fit the Program easily into their lives and the spaces in which they lived; they made progress towards what mattered, and in their lives; and they could drive their recovery by building their own plan and recognising their efforts. Descriptions of each category and corresponding quotations are provided in Table 6.

**TABLE 6:** Acceptability of the Living My Life Program (*n* = 4).

Categories	Sub‐categories	Findings	Quotations
I'm fitting it in easily	*Working within my lifestyle*	The Program fitted into participants' lifestyles and the spaces in which they lived. Scheduling sessions around commitments and linking in with local personal and professional support networks helped, as did using technologies they already had access to. Making plans that accommodated their preferences and that slotted into their life meant they could adjust their plans along the way.	*I don't have to step outside of my life to do this rehab thing it fits really well* [P1]
	*Working in my space*	Committing to the 12‐week Program created space in participants' lives to think about what mattered to them; to see progress; to build habits; and to feel ready to go it alone by the end. Activities that didn't fit easily could be set aside, as were technologies that were hard to get going with or no longer needed. Taking pressure off helped if plans became overwhelming, amongst the demands of life, injury or illness, or the participant's own expectations. Anchoring the Program in their life meant participants didn't disrupt their usual routines to work towards what mattered.	
I'm making progress	*Working on what matters to me*	Working on goals grounded in their daily lives made their program feel relevant. Sensing improvement, participants used everyday technologies to check their progress. Seeing improvements in their goals and their lives motivated them to keep working towards what mattered, even after the Program finished.	*the videos mean more to me because I can see my progress. I can't explain everything in a number*. [P1]
	*Living my life*	Achieving goals inspired them to explore other activities, sometimes contemplating activities that they previously considered unimaginable. While continuing to live their lives, participants attributed the progress they made to driving their own recovery.	
I'm driving my recovery	*Owning my plan*	Participants gained confidence in driving their recovery by building their own action plans and recognising that their efforts led to progress. Building their plan meant setting the direction, then designing and moulding it to fit within their lives.	*you hit a problem and I can always come up with a possible solution. And then, if I got somebody that I can ask, they can tell me, whereas before it'd be, leave them to sort out what needs to be done*. [P3]
	*Putting in the effort*	Feeling accountable made them ‘put in the effort’ initially. Recognising results came from their efforts motivated them to keep going, even on days that seemed harder. They were increasingly adapting their plans to work in with their life and as they made progress. Yet, confidence could diminish if there were concerns about safety or uncertainty about activities. Participants felt comfortable to seek help from health professionals in these instances. Developing confidence in using technology excited some participants, opening new doors while reducing their reliance on those around them for technology support. Participants' confidence to drive their recovery was reinforced by seeing progress, and the ease with which they fitted the Program within their lives.	

## 8 Discussion

The Living My Life Program was feasible and acceptable to a small, diverse group of rural and remote stroke survivors. They were able to fit the Program into their lives, allowing them to drive their recovery and make progress towards what mattered to them. In essence, the Program enabled them to recover their way, in their world.

From the stroke survivors' perspective, the Program was feasible because it became part of their everyday lives, through flexible online sessions, building their own plans, and using everyday technologies. Fitting rehabilitation into their ever‐changing lives contrasts with the usual experience for rural and remote stroke survivors, who often disrupt their lives to participate, e.g., travelling away from community, family and cultural connections [1, 3, 34] or taking time off work [2]. Disruption can lead to disengagement, e.g., when rehabilitation is considered not worth the effort or inconvenience [3, 34], or contrary to one's own cultural beliefs [1]. For the stroke survivor who did not complete the program, the requirement of going to a public facility with adequate internet access and at a mutually agreeable time, may have contributed. Confirming the findings of previous studies [3, 34], persevering with recovering was made easier because the Program fitted into the stroke survivors' everyday lives, enabling them to continue recovering as their life evolved.

Technologies used by the stroke survivors were for the most part those they already had access to, making them convenient and relevant in their life. Consequently, most stroke survivors had the resources and support they needed to participate, adjusting how they used everyday technology to work for them. The stroke survivor who did not complete the Program was the exception. For rural and remote stroke survivors in Australia, studies that have investigated use of technology [10] have generally focused on technologies prescribed by a service rather than chosen by the stroke survivor. The current study findings reinforce the view that technology can offer some stroke survivors greater choice and control over their rehabilitation [35] through the use of everyday technologies [36]. Rather than restricting stroke survivors to prescribed technologies, the importance of incorporating everyday technologies according to what works for them, in their world, cannot be overstated [2]. To ensure an individual's choice is not limited by their own environment, challenges of access to technology and necessary supports must be addressed [8, 35].

Measuring progress towards what mattered for each stroke survivor using a combination of PROMs, complemented by videos and feedback from apps, was feasible. As previously reported [3], having goals and seeing meaningful progress helped these stroke survivors persevere. If progress was interrupted or stalled (e.g., by everyday life), practice was postponed or modified, rather than abandoned, until circumstances improved. Understanding how stroke survivors determine progress is critical to understanding whether recovery has been achieved. Similar to previous findings [3], meaningful progress translated into doing everyday activities. Standardised questionnaires (e.g., Lawton Instrumental Activities of Daily Living [37]) do not always reflect meaningful change for individual stroke survivors, nor their evolving aspirations and perception of their recovery potential [12]. Recognising that PROMs are subjective, and may be influenced by goal‐setting and changing circumstances [38], further investigation is required to determine the ideal combination of measures that enable services and stroke survivors to capture meaningful progress, potentially using everyday technologies.

Acceptability of the Program, by those who completed it, was high because they felt the Program worked for them. This is perhaps not surprising, given that stroke survivors and an expert panel contributed to its development [39]. Furthermore, the findings fit with stroke survivors' views of: setting rehabilitation goals that matter [3, 34]; being consulted when making plans [34]; sharing positive interactions with providers [1, 34]; and incorporating technology in these processes [38]. Stroke survivors planning their program in this way is consistent with exercising individual choice and control, a core principle of the NDIS, which is Australia's primary mechanism for funding disability supports [9]. Aligning with funding mechanisms is an important consideration for translating the Program into practice.

## 9 Strengths and Limitations

Feasibility and acceptability were established with a group of stroke survivors motivated to improve recovery for themselves and for others, which is often the case in research [40]. Those who chose not to volunteer, who were perhaps not inclined to continue with rehabilitation or to use technology, may hold different opinions. Similarly, positive feedback provided may reflect the view that any service is better than no service when there are limited options for rehabilitation available locally [6, 8].

The dual role of the stroke survivor as co‐researcher provided a continual reminder of the stroke survivors' lens. The dual role of the coach and lead researcher provided continuity leading to open, authentic communication. Conversely, this duality may have influenced participants' engagement and their responses during evaluation, positively or negatively. Recognising this limitation, strategies (e.g., member checking, discussions with expert panel) to enhance rigour during data collection and analysis were used.

## 10 Future Implications

Continuing with an action research approach, the next step is to offer the Program to stroke survivors: earlier in the care pathway; from diverse cultures; or with varied stroke‐related impairments and severity delivered through existing services or funding programs (e.g., NDIS).

Priority must be given to ensuring the Program is feasible and acceptable for Aboriginal and Torres Strait Islander Peoples, given their high incidence of stroke and at young ages [41]; and the opportunity this Program offers to increase their engagement in rehabilitation in light of systemic challenges [1]. Recognising diversity within and between language groups, future research should prioritise connections with local networks and cultural mentors to ensure the research aligns with ethical guidelines [42]. Time and support to establish and maintain connections should be factored into broader implementation [43]. Piloting within a culturally responsive service that is well‐connected in the community is recommended [44, 45].

## 11 Conclusion

The Living My Life Program was feasible and acceptable for the stroke survivors from rural and remote locations. Using everyday technologies, they made progress towards what mattered to them and were able to drive their recovery without disrupting their lives. Further research is required to determine the Program's broader applicability, particularly with stroke survivors from diverse cultures and with varying stroke‐related impairments and severity.

## Author Contributions

**Sarah M. Jackson:** conceptualisation, data curation, formal analysis, funding acquisition, investigation, methodology, project administration, resources, visualisation, writing – original draft. **Lucy M. Byrnes:** investigation, visualisation, writing – review and editing. **Shaun D. Solomon:** conceptualisation, investigation, visualisation, writing – review and editing. **Bridee A. Neibling:** investigation, writing – review and editing. **Louise F. Brown:** investigation, writing – review and editing. **Ian M. Atkinson:** investigation, supervision, writing – review and editing. **Ruth N. Barker:** conceptualisation, funding acquisition, investigation, supervision, visualisation, writing – review and editing. All authors approved the submitted manuscript.

## Ethics Statement

The trial was approved by the James Cook University Human Research Ethics Committee (H8616) and registered with the Australian New Zealand Clinical Trials Registry.

## Conflicts of Interest

The authors declare no conflicts of interest.

## Supporting information


**File 1** Interview topic guide for the Living My Life Program.

**File 2** Acceptability of Intervention Measure (AIM) [28] for the Living My Life Program (*n* = 4).

## Data Availability

Data generated in the current study are not publicly available due to privacy and HREC restrictions. Deidentified quantitative data are available from the corresponding author upon reasonable request.
