# Supplementary material for: Feasibility and Acceptability of the Living My Life Program for Rural and Remote Stroke Survivors
Source: Aust J Rural Health. 2026 Apr 7;34(2):e70166. doi: 10.1111/ajr.70166 (PMC13055121; doi:10.1111/ajr.70166)
Supplement: Supplementary file 1 — File 1 Interview topic guide for the Living My Life Program. [file AJR-34-0-s001.docx]

Supplementary file 1. Interview topic guide for the Living My Life Program.

| **Questions** |
| --- |
| 1. How is life going for you? 2. How did you find the Program? 3. How relevant is the Program to living your life? 4. How did the Program fit with recovering your way? 5. How did the Program fit with recovering in your world? 6. How did you find:    1. ‘working out what matters’ (goal setting)?    2. ‘working towards what matters’?       1. Co-designing your action plan       2. Doing your action plan       3. Coaching sessions with the therapist       4. Using your logbook       5. Using your journal       6. ‘Measuring’ your progress       7. Thinking about your future (setting goals and action plan) 7. How did you find using technology in the Program? 8. How did you find fitting the Program in with living your life? 9. Would you recommend this program to others? 10. Would you complete this program again? 11. So, in summing up, how acceptable was the Living My Life Program to you? (Refer to AIM survey results) |
| *AIM* Acceptability of Intervention Measure^28^. |
