# Supplementary material for: Feasibility and Acceptability of the Living My Life Program for Rural and Remote Stroke Survivors
Source: Aust J Rural Health. 2026 Apr 7;34(2):e70166. doi: 10.1111/ajr.70166 (PMC13055121; doi:10.1111/ajr.70166)
Supplement: Supplementary file 2 — File 2 Acceptability of Intervention Measure (AIM) [28] for the Living My Life Program (n = 4). [file AJR-34-0-s002.docx]

Supplementary file 2. Acceptability of Intervention Measure (AIM)^28^ for the Living My Life Program (n = 4).

| **Statement** | **Median** | **Range** |
| --- | --- | --- |
| 1. The Living My Life Program meets my approval. | 5 | 5 |
| 2. The Living My Life Program is appealing to me. | 5 | 4 – 5 |
| 3. I like the Living My Life Program. | 5 | 4 – 5 |
| 4. I welcome the Living My Life Program. | 5 | 5 |
